# Development of a group structured education programme to support safe exercise in people with Type 1 diabetes: the EXTOD education programme

**DOI:** 10.1111/dme.14064

**Published:** 2019-07-31

**Authors:** P. Narendran, S. Greenfield, J. Troughton, Y. Doherty, N. Quann, C. Thompson, I. Litchfield, R. C. Andrews, the EXTOD Education Programme Development Team

**Affiliations:** ^1^ Department of Diabetes University Hospitals Birmingham NHS Foundation Trust Birmingham; ^2^ Institute of Immunology and Immunotherapy University of Birmingham Birmingham; ^3^ Institute of Applied Health Research University of Birmingham Birmingham; ^4^ Leicester Diabetes Centre University Hospitals Leicester Leicester; ^5^ Department of Psychological Medicine York Teaching Hospitals NHS Foundation Trust York; ^6^ Leicester Clinical Trials Unit College of Life Sciences University of Leicester Leicester; ^7^ Department of Diabetes Taunton and Somerset NHS Foundation Trust Taunton; ^8^ University of Exeter Medical School Exeter UK

## Abstract

**Aim:**

To develop a structured education programme for individuals with Type 1 diabetes who are engaging in regular exercise.

**Method:**

A multidisciplinary team of experts in supporting exercise and physical activity for people with Type 1 diabetes, alongside researchers with experience of developing self‐management education, developed an exercise programme using the Medical Research Council framework. The programme was informed by a review of the evidence relating to Type 1 diabetes and exercise, the behaviour change literature (including the behaviour change taxonomy), and qualitative interviews with stakeholders. The programme and supporting resources were refined using an iterative process of testing, delivery and collecting feedback from participants and the wider development team.

**Results:**

The outcome of the intervention development was the design of a feasible and acceptable intervention for people with Type 1 diabetes to support safe exercise. The pilot allowed refinement of the intervention prior to testing in a two‐site feasibility randomized controlled trial. Key findings from the pilot informed minor restructuring of the timetable (timings and order) and adaptation of supporting educational materials (participant handbook and teaching materials).

**Conclusion:**

The ‘EXercise in people with Type One Diabetes’ (EXTOD) education programme has been developed using robust methodology for the generation of educational interventions. It now needs testing in a randomized controlled trial.

## What's new?

- Currently there is no dedicated structured education programme that supports the management of Type 1 diabetes for exercise.
- A self‐management education programme to support safe and effective exercise for people with Type 1 diabetes has been developed using the Medical Research Council framework.
- In the exercise programme we developed, participants were able to adjust their regimens and plan their exercise in a more structured way.
- The programme was delivered by trained healthcare professionals, meaning it can be embedded into the specialist care setting.
- If demonstrated to be effective, this programme could potentially expand currently available support for adults with Type 1 diabetes who wish to exercise.

**What's new?**

- Currently there is no dedicated structured education programme that supports the management of Type 1 diabetes for exercise.
- A self‐management education programme to support safe and effective exercise for people with Type 1 diabetes has been developed using the Medical Research Council framework.
- In the exercise programme we developed, participants were able to adjust their regimens and plan their exercise in a more structured way.
- The programme was delivered by trained healthcare professionals, meaning it can be embedded into the specialist care setting.
- If demonstrated to be effective, this programme could potentially expand currently available support for adults with Type 1 diabetes who wish to exercise.

## Introduction

Participation in regular exercise in people with Type 1 diabetes improves physical fitness and strength, reduces cardiovascular risk factors and improves well‐being 1. It also reduces insulin requirements and improves insulin resistance as well as reducing cardiovascular disease and mortality 1. Based on this evidence, Diabetes UK and the American Diabetes Association recommend people with Type 1 diabetes undertake at least 150 min per week of moderate to vigorous aerobic exercise with two to three sessions per week of resistance exercise on non‐consecutive days 2.

Glucose levels during exercise are under the physiological control of insulin as well as counter‐regulatory hormones such as glucagon, growth hormone, cortisol and catecholamines 3, 4. The secretion of these hormones changes before, during and after exercise to facilitate recovery and adaptation to exercise. In Type 1 diabetes, the insulin level does not fall in response to exercise and there may be impaired secretion or action of counter‐regulatory hormones, making normal fuel regulation difficult 5. People with Type 1 diabetes, therefore, need to make carefully calculated changes in insulin dosage and nutritional intake in line with the normal expected physiological responses to the particular exercise and intensity they are performing. This requires knowledge and skills.

In a study of 2185 people with Type 1 diabetes, from 16 European countries, 786 participants (36%) engaged in none, or only mild physical activity 6. In the Finnish Diabetic Neuropathy Study, 23% of people with Type 1 diabetes were found to be sedentary and a further 21% participated in less than one episode of exercise per week 7. We have recently measured objective levels of exercise in adults with Type 1 diabetes and demonstrated that they spend a quarter less time in moderate to vigorous physical activity per day than healthy adults 8. These findings indicate that a high percentage of people with Type 1 diabetes are insufficiently active.

Reported barriers to exercise in those with Type 1 diabetes include fear of hypoglycaemia, competing work commitments, loss of control over diabetes, low fitness levels, cost, lack of social support, and lack of knowledge 3, 4. Greater knowledge about insulin pharmacokinetics and using appropriate approaches to minimize exercise‐induced hypoglycaemia or hyperglycaemia are factors associated with fewer perceived barriers to exercise 3, 4. There is also a growing evidence base that a multitude of psychosocial factors including environment, social norms, beliefs and emotions can influence the ability of a person with Type 1 diabetes to manage their condition in order to achieve satisfactory outcomes whilst retaining psychological well‐being 9, 10, 11. This suggests that the provision of knowledge and skills is essential if we are to support people with Type 1 diabetes to become more active and enable them to exercise safely at the level they wish to.

The National Institute for Health and Care Excellence (NICE) recommends that all people with Type 1 diabetes receive advice and education about exercising safely with Type 1 diabetes as part of their routine care, from diagnosis onwards 12. They also recommend that all people with Type 1 diabetes have access to structured educational programmes as an integral part of their care pathway.

Structured education refers to group‐based patient‐centred educational programmes that have a clear philosophy and a written curriculum with supporting resources, are underpinned by appropriate learning and psychological theory, and are evidence‐based and delivered by trained, quality‐assessed educators. In this article we describe the development, testing and refining of a structured education programme for adults with Type 1 diabetes who are already undertaking regular exercise. This programme was developed using the Medical Research Council framework 5 and intervention mapping 6 so that it can be fit for purpose for formal evaluation in a feasibility randomized controlled trial (RCT). The education programme has been termed the ‘EXercise in people with Type One Diabetes (EXTOD) Education’ programme.

## Methods

**Figure 1:**
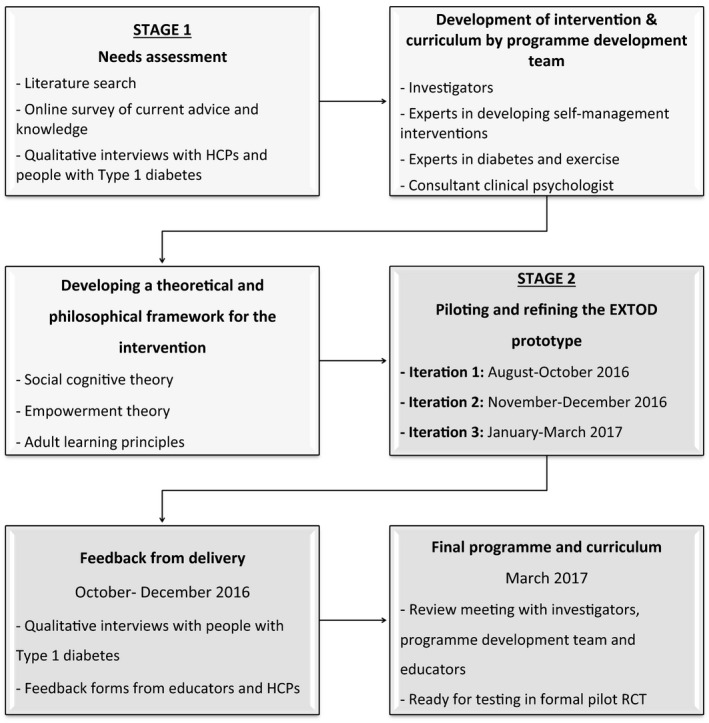
Stages of development for the EXTOD education programme. HCP, healthcare professional; RCT, randomized controlled trial.

The intervention development took place as the first phase of a National Institute for Health Research (NIHR) Research for Patient Benefit project (PB‐PG‐1013‐32096). Ethical approval was granted by West Midlands ‐ Coventry and Warwickshire Research Ethics Committee (reference 16/WM/0034; IRAS ID 178659). The development process for the EXTOD education programme took place over 23 months between July 2015 and July 2017. The education curriculum was then ready for testing in a feasibility RCT. An overview of the stages of development is shown in Fig. 1.

The EXTOD education programme was developed by a multidisciplinary team consisting of clinical experts in the field of supporting exercise for people with Type 1 diabetes (consultant diabetologists, diabetes dietitians, diabetes nurses), and researchers with experience of developing self‐management education. The teams were based at three sites in the UK (Taunton, Birmingham and Leicester) and communicated regularly to share knowledge, discuss ideas and reach consensus.

The EXTOD programme was designed to be a group educational programme with a written curriculum and supporting educational resources suitable for a broad range of participants with Type 1 diabetes already engaging in regular physical activity. It was developed for participants who were on a basal–bolus insulin regimen, were prepared to monitor their blood glucose levels, had the ability to carbohydrate‐count and had a basic understanding of the principles of insulin dose adjustment. The programme was designed to be delivered by trained educators, nationally accredited in delivering structured education to people with Type 1 diabetes.

### Needs assessment

To begin developing the prototype for EXTOD education, a needs assessment was carried out. This consisted of a literature review of the evidence, a survey of current practice, and qualitative work (Fig. 1).

The HCPs were asked to complete an online survey so that we could collect data on current practice regarding the education of people with Type 1 diabetes regarding activity and identify education gaps. The survey was disseminated through national medical, nursing and dietetic HCP organizations in order to obtain information from as many HCPs as possible. A total of 127 doctors, 93 dietitians, 65 nurses and 40 physiotherapists responded (325 HCPs in total).

This was followed by focus groups and semi‐structured interviews conducted with people with Type 1 diabetes and HCPs (dietitians, diabetes nurses and diabetologists) likely to be delivering the new education programme. The methodology for this qualitative work is reported separately 13.

### Developing a theoretical and philosophical framework for the intervention

A theoretical framework was developed using the recommended processes 14, 15 and any theoretical underpinnings used in other interventions identified by the literature review.

A set of problem behaviours and target behaviours was identified specific to people with Type 1 diabetes wishing to become more confident in exercise. These were refined by consideration of the literature and the qualitative results and discussion amongst the wider team 16. This, in turn, informed identification of the specific behaviour change techniques that would be employed, the theory and the structure, format and delivery of the programme. In addition the theoretical framework clarified the behaviours to be enacted by the EXTOD educators and informed development of the treatment fidelity tool used to observe these behaviours in the delivery stage of the research. In accordance with good practice guidelines 12 a guiding philosophy was developed by the education team and shared, debated and agreed by the wider collaborative team.

The development team, having established the theoretical and philosophical framework of the intervention, took the learning and insights from the qualitative work and combined this into the EXTOD education prototype intervention. This comprised stage one of the development.

### Testing and refining the EXTOD education prototype

Stage two of the programme development involved a cycle of three iterations of delivery of the EXTOD education prototype to groups of participants with Type 1 diabetes, followed by evaluation, modification and retesting of the prototype. Iterations were conducted at two hospitals in the UK: Taunton and Somerset NHS Foundation Trust, which is a medium‐sized hospital in the Southwest that provides care for people living in a mixture of rural and urban environments, and the University Hospitals Birmingham NHS Foundation Trust. The latter is a large teaching hospital in the West Midlands that provides care for people living predominantly in an urbanized metropolitan environment. Experiential learning of developers, participants and educators from each delivery event, was fed back to produce the next version of the intervention.

The first iteration was conducted in Taunton between August and October 2016. The prototype was delivered to a group of people with Type 1 diabetes by the educational researchers from Leicester, and was observed by a maximum of three HCPs at a time, as part of their training to become prospective educators. At each site, HCPs trained to deliver EXTOD included a diabetologist, nurse and dietitian who were all experienced in delivering structured education to people with Type 1 diabetes. Feedback from the people with Type 1 diabetes and observing HCPs was collected and used to refine the intervention.

The second iteration took place in Birmingham between November and December 2016. The prototype was delivered to a fresh group of people with Type 1 diabetes by the educational researchers from Leicester, and observed by different HCPs. The training programme was developed in parallel with the curriculum, and was informed by the content, the theoretical underpinning of EXTOD.

The third iteration was delivered by the new educators to two further groups of participants with Type 1 diabetes in Taunton and Birmingham between January and March 2017, delivering the revised intervention. The iterations were observed and feedback was provided on the accuracy of the content.

After completion of three cycles of piloting at each site, there was a final refinement of the educator curriculum and all educational material to ensure it was fit for purpose for use in a feasibility randomized controlled trial..

### Participants and recruitment

Study inclusion criteria included a clinical diagnosis of Type 1 diabetes, age 18–70 years, hypoglycaemia awareness, knowledge of carbohydrate‐counting, being on a basal–bolus regimen, completion of a nationally accredited structured education programme for people with Type 1 diabetes, such as Dose Adjustment for Normal Eating (DAFNE) 17 or local equivalent such as Beta Cell Education Resources for Training in Insulin and Eating 18, and doing at least 30 min of exercise twice a week or being signed up to a sporting event to take place in the next 3–6 months. Participants were excluded if they were pregnant, using an insulin pump, had hypoglycaemia unawareness, or were unable to exercise, understand English or give informed consent. People using insulin pumps were excluded from the present study because the approaches to managing insulin adjustments around exercise differ from those required when using insulin pens.

### Data collection

For each iteration, a range of methods was used to evaluate the prototype including observations, one‐to‐one interviews and questionnaires. Each session was filmed with the consent of the participants, enabling researchers to review and evaluate the prototype content, resources and delivery style of the educators. Additional notes were taken by researchers at each session, plus timings of each session and total timing of the programme.

## Results

### Stage one results

#### Qualitative findings

Findings from the four focus groups with people with Type 1 diabetes and with HCPs conducted at the Taunton and Birmingham sites are reported separately 13. We found that the successful provision of education and advice was influenced by factors relating to the individual with diabetes and their service provider. Factors related to the individual with diabetes included the type of activity and complexity of the exercise regimen, the level of engagement with his/her condition and care and health literacy. Service‐related factors included inconsistent training, a lack of capacity and continuity and limited coherence of information from across their care team 11.

### Theoretical framework

The theories and models identified from the literature review as most likely to increase confidence in people with Type 1 diabetes to manage their exercise of choice safely were social cognition theory 19 an empowerment approach 20 and a heuristic‐systematic model 21 (Table S1).

#### The prototype EXTOD programme

The EXTOD prototype was designed to consist of three workshops totalling 9.5 h (4 h, 3 h and 2.5 h, respectively; Table 1) to be delivered to small groups of eight to 12 participants over an 8‐week period, by three trained educators.

**Table 1:** Final EXTOD education programme outline

Session 1 Total time 4 h	Session 2 Total time 3 h	Session 3 Total time 2.5 h
Welcome (10 min)	Welcome Back (5 min)	Welcome Back (5 min)
Where Are You Now? (40 min)	Sharing Stories (40 min)	Sharing Stories (40 min)
*Identifying personal experiences, expectations and goals when exercising with Type 1 diabetes*	Participants feedback their experiences since session 1	Participants feedback their experiences since session 2
	Understanding Your Mechanics 2 (30 min)	Advanced Strategies (80 min)
		*Putting all the ICE strategies together and applying to complex scenarios using case studies*
	*Glucose regulation in the body after exercise*	
Understanding Your Mechanics 1 (80 min)	Fuel for Exercise (60 min)	
	*Nutrition for effective exercise and glucose control*	
*Glucose regulation in the body at rest and during varying exercise types in someone with and without Type 1 diabetes*		
Staying Safe (30 min)	Strategies after exercise (30 min)	
*Using an algorithm to determine safe limits for starting exercise how to treat or prevent hypoglycaemia (hypo)*.		
	*Discussion of application of ICE strategies to control glucose levels after exercise*	
*Exercise and complications of diabetes*.		
*Staying Safe Checklist*		
Strategies before and after exercise (60 min)	Next Steps (15 min)	
*ICE Strategies to manage blood glucose before and during exercise*	*Identifying goals for the next month, completing a written action plan and sharing with the group*	
*Insulin adjustments, Carbohydrate for exercise and adapting type or order of Exercise*		
Next Steps (20 min)		Future Planning (25 min)
		*Making an action plan for next steps, building on what has been learnt from the course*.
*Making a personal action plan and sharing it with the group*		

ICE, insulin, carbohydrate and exercise.

The key learning opportunities for participants attending the programme were as follows.

**Figure 2:**
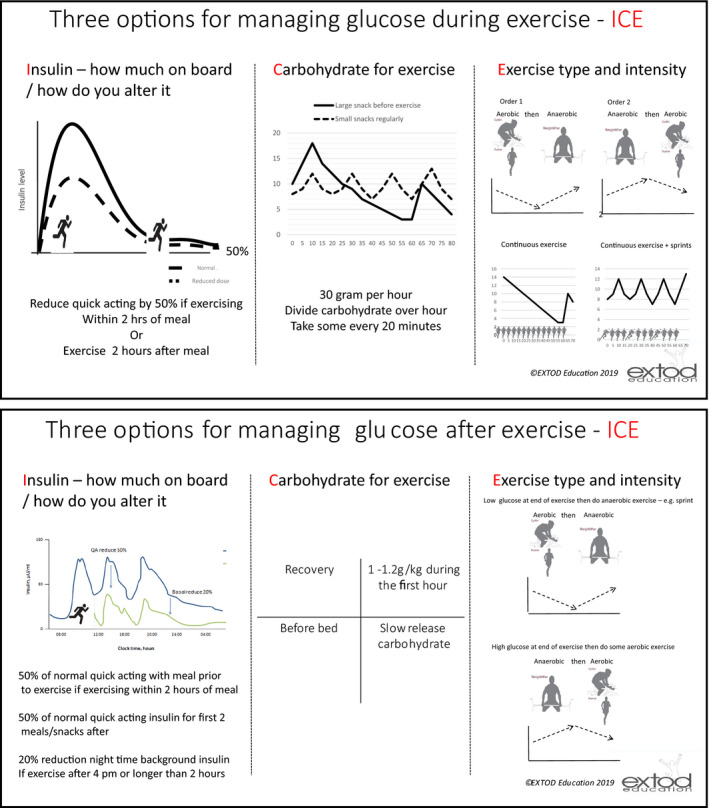
Summary diagrams of insulin, carbohydrate and exercise (ICE).
- To test out ICE strategies using a stepwise approach.
- To develop problem‐solving skills in relation to managing safe exercise, specifically identifying barriers, considering solutions, engaging in a plan and testing it out.
- To learn from each other ways to overcome the challenge of exercising with Type 1 diabetes by sharing stories.
- To explore and develop a plan for overcoming future potential relapse and maintaining motivation.

- To consolidate core knowledge and skills in carbohydrate‐counting, monitoring and insulin adjustment, and management of hypoglycaemia.
- To learn new exercise strategies for adjusting insulin‐, carbohydrate‐ and exercise‐ ‐ordering (referred to as ICE within the programme). These ICE strategies were developed for EXTOD based on the consensus statement on exercise for Type 1 diabetes 22 (examples provided in Fig. 2).

### Stage two results

Stage 2 involved the piloting and refining of the protocol through three different iterations.

#### Recruitment and attendance at EXTOD sessions

People with Type 1 diabetes were recruited to participate in three iterations of the programme, each undertaken at Birmingham and Taunton. The consort diagram outlining recruitment and attendance is shown in Fig. [Link].

#### Characteristics of participants

Of the 28 participants who attended the programme at both sites, 15 were women (54%), and 13 (46%) were men. More women (*n* = 12) than men (*n*= 7) attended in Taunton, and more men (*n*= 6) than women (*n*= 3) attended in Birmingham. The mean age was 45 years. Women attendees in Taunton tended to be younger than in Birmingham (the youngest at Taunton was 26 years, the youngest at Birmingham was 52 years); however, the age range of men was similar across both sites (Table S2).

#### Feedback from iterations of the education programme

After completion of the education iterations, interviews were conducted with eight participants by a qualitative researcher on 12 October 2016 and 14 December 2016. Educators and HCPs were also provided with feedback forms to reflect on their experience of observing the sessions. The key themes generated from these interviews and feedback forms related to: the format and timing of the programme; the content of the programme; the use of resources and the use of medical jargon.

Overall, participants believed the programme afforded them the opportunity to test the strategies and gain confidence to exercise safely. Participants reported that they had been able to adjust their regimens in line with their exercise and that the programme had helped them to ‘build up the confidence to exercise at different times of the day’ and plan exercise in ‘a more structured way, in a more mathematical way’. Some members of the group had been ‘hoping for something like this for a long time’ and wished they ‘had known about it 20 years ago’. One participant felt that prior to attending the education sessions, they were aware of the symptoms of hypoglycaemia, but ‘did not really know what was happening to [their] body and why it was happening, but now [they] understand… it makes it so much easier’.

The key learning points as reported by participants were:

- the three ways to manage exercise, and realising there is not just one solution;
- becoming confident in insulin adjustment;
- strategies to plan for exercise;
- different types of exercise and the impact on blood glucose levels.

#### The final programme

The final EXTOD programme was agreed upon after a review meeting with the Chief Investigator, Principal Investigator, the Education Development Team from Leicester and trained educators from Birmingham, Taunton and Leicester, held on 29 March 2017. The final format is outlined in Table 1.

## Discussion

In the present paper we describe the development of the first group‐based structured education programme specifically designed for people with Type 1 diabetes to enable them to manage their exercise choices confidently and safely. Currently, there is no other dedicated, structured education programme that aims to support people with Type 1 diabetes to manage their diabetes for exercise. DAFNE, a structured education programme for Type 1 diabetes 17, currently dedicates 60 min to physical activity, but anecdotal evidence suggests people with Type 1 diabetes do not find this sufficient. Information also exists in the form of leaflets and reviews, but again these do not involve HCPs and people with Type 1 diabetes in discussing the principles involved in managing Type 1 diabetes for exercise.

The EXTOD education programme has been developed specifically to meet the National Guidance for a Structured Education Self‐management Programme 16, being grounded in an evidence base, underpinned with a philosophy and theory previously demonstrated to promote behaviour change and successfully used in other validated structured self‐management programmes for diabetes. The intervention itself was delivered by trained and quality‐assured educators, meaning this programme can be embedded into the specialist care setting. Key to this programme's success was the attention given to educator training and the iterative development of the curriculum and supporting resources.

It is important to recognize the range of psychosocial factors which may impact on the ability of a person with Type 1 diabetes to satisfactorily self‐manage, both from the perspective of clinical outcomes and individual well‐being. There are also many barriers for people with Type 1 diabetes to integrate their diabetes, specifically with regard to exercise, into their daily life 4, 22, 23.

A strength of this programme is that it is based on extensive needs analysis of people with Type 1 diabetes and of HCPs, a review of current evidence, and the experiences of HCPs involved in supporting people with Type 1 diabetes to exercise. It has been developed based on the Medical Research Council framework for developing and evaluating complex interventions 5 and intervention mapping 6. Our iterative and reflective process, supplemented by qualitative research methodology, is a tried and tested approach previously used successfully by our group 5. Further strengths of this programme are that it included a multidisciplinary team and shared learning, and provided a forum for identifying learning needs or gaps in knowledge in a safe group setting. The challenges were that learning required working of teams across three separate sites and developing new learning and new ways of working.

The resulting EXTOD education programme is now being tested and evaluated in a formal feasibility RCT in which participants will be randomized to receive the education programme or usual care. The formal feasibility RCT will help to determine whether the EXTOD Education programme increases exercise, reduces glucose variability around exercise, and addresses the barriers to exercise in people with Type 1 diabetes. If demonstrated to be effective, this programme has the potential to expand current practice for adults with Type 1 diabetes.

## Funding sources

The EXTOD education programme was funded by the NIHR Research for Patient Benefit programme. The study was supported by the University of Leicester Clinical Trials Unit.

## Competing interests

All the authors declare that they have no competing interest, financial or otherwise. The views expressed are those of the author and not necessarily those of the NHS, the NIHR or the Department of Health and Social Care.

## Ethical approval

The study was approved by the West Midlands ‐ Coventry and Warwickshire Research Ethics Committee (REC reference 16/WM/0034; IRAS ID 179659), and informed consent was obtained from all participants. This research study was conducted in accordance with the guidelines of the Declaration of Helsinki.

## Supporting information


**Figure S1.** Consort diagram for overall education iterations.
**Table S1.** Theoretical framework underpinning EXTOD.
**Table S2.** Characteristics of participants attending EXTOD Education course.

**Appendix S1.** EXTOD– participant interview topic guide for feedback.

**Appendix S2.** EXTOD– educator feedback.
