# Supplementary material for: Development of a group structured education programme to support safe exercise in people with Type 1 diabetes: the EXTOD education programme
Source: Diabet Med. 2019 Jul 31;37(6):945–52. doi: 10.1111/dme.14064 (PMC7317834; doi:10.1111/dme.14064)

**Supplementary table 1:** Theoretical framework underpinning EXTOD

| **Problem behaviours** | **Potential behavioural outcome (Target behaviours)** | **Theory** | **Mapping to behavioural taxonomy (**[**24**](#_ENREF_24)**,** [**25**](#_ENREF_25)**)** | **Sample activity within EXTOD programme** |
| --- | --- | --- | --- | --- |
| Low levels of confidence to engage in physical activity of choice | High levels of confidence to engage in physical activity of choice | **Self-efficacy (**[**26**](#_ENREF_26)**,** [**27**](#_ENREF_27)**)**   - **Mastery** (previous successful attempts of the behaviour) - **Modelling** (observing others like oneself engaging in the behaviour) - **Verbal persuasion** (talking through the process of change, planning for obstacles, success) - **Emotions management** | - Focus on past successes - Self-monitoring of behaviour outcomes and consequences - Instruction on how to perform behaviour - Graded tasks - Behavioural experiments - Credible source - Habit reversal - Review behavioural goal - Social comparison | **Sharing stories sessions:**   - Eliciting what has gone well in terms of behaviour change, problem solving around challenges and observing others successes and challenges - Discussion of barriers to change - Acknowledgement of feelings and emotions   **Next steps and future planning sessions**   - Action planning - Problem solving - Setting short term goals |
| Not measuring blood glucose (BG) at appropriate times, before, during and after exercise. | Taking blood glucose (BG) measurement at the times appropriate to exercise. | Social Cognitive theory **(16)** | - Behavioural substitution - Habit reversal - Self-beliefs | Exploration of barriers and enablers to BG monitoring at specific times  Facilitation of individualised problem solving strategies. |
| Not carrying hypo treatments and/or having incorrect hypo treatments | Deciding on appropriate hypo treatment and taking it to all exercise activities. | Social Cognitive theory **(16)** | - Behavioural substitution - Habit reversal | Supporting the exploration of barriers and enablers to carrying the correct hypo treatment  Facilitation of individualised problem solving strategies |

| **Problem behaviours** | **Potential behavioural outcome (Target behaviours)** | **Theory** | **Mapping to behavioural taxonomy (**[**24**](#_ENREF_24)**,** [**25**](#_ENREF_25)**)** | **Sample activity within EXTOD programme** |
| --- | --- | --- | --- | --- |
| Continuing to exercise following a low/high BG reading. | Taking necessary precautions if BG readings are outside of safe zone – delaying exercise or making corrections and retesting before commencing exercise. | Social cognitive theory **(16)** | - Behavioural substitution - Habit reversal | Supporting the exploration of barriers and enablers to exercising when BG are too high or too low  Facilitation of individualised problem solving strategies |
| Not considering the longer-term influence of exercise on BG levels and therefore not taking steps to avoid hypos up to 14 hours after exercise. | Having a plan to ensure BG does not drop too low – meals, snacks, bedtime snack to protect against hypo. | Social cognitive theory **(16)** | - Problem solving - Goal setting - action planning - Anticipated regret | Supporting the exploration of barriers and enablers to longer term BG monitoring  Facilitation of individualised problem solving strategies |

**Supplementary table 2:** Characteristics of participants attending EXTOD Education course

| **Characteristics of participants attending EXTOD Education course** | | |
| --- | --- | --- |
| **Taunton** | | |
| Women | 12 | 18-64 |
| Men | 7 | 24-68 |
| **Birmingham** | | |
| Women | 3 | 52-69 |
| Men | 6 | 28-60 |
| **Total** | | |
| Women | 15 | 19-69 |
| Men | 13 | 24-68 |

**Supplementary fig. 1:** Consort diagram for overall education iterations


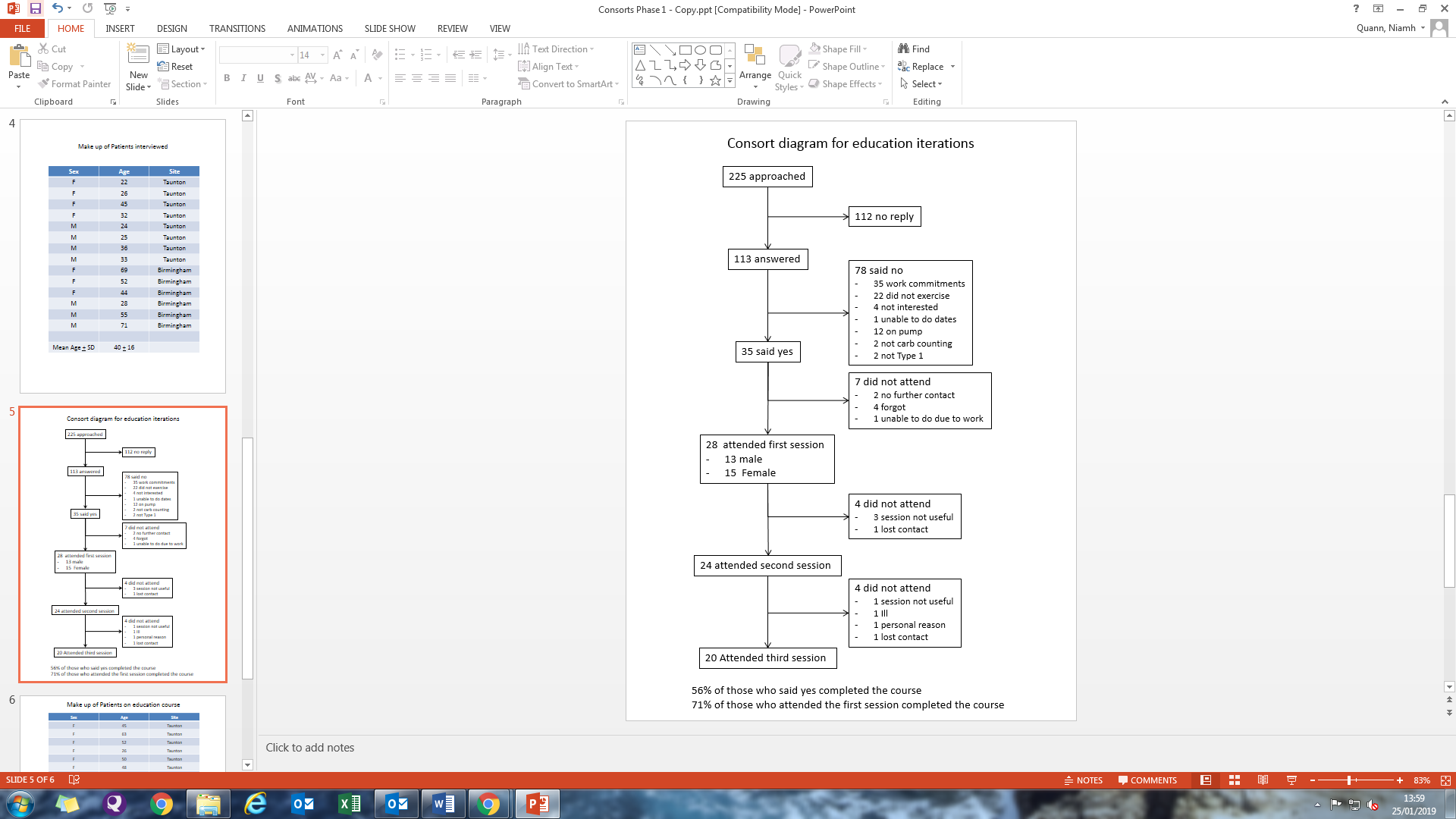

Supplement: Supplementary file 1 — Figure S1. Consort diagram for overall education iterations. Table S1. Theoretical framework underpinning EXTOD. Table S2. Characteristics of participants attending EXTOD Education course. [file DME-37-945-s001.docx]
