# Supplementary material for: Development of a group structured education programme to support safe exercise in people with Type 1 diabetes: the EXTOD education programme
Source: Diabet Med. 2019 Jul 31;37(6):945–52. doi: 10.1111/dme.14064 (PMC7317834; doi:10.1111/dme.14064)
Supplement: Supplementary file 2 — Appendix S1. EXTOD– participant interview topic guide for feedback. [file DME-37-945-s002.docx]

**Appendix 1:** Participant interview Topic guide for feedback

**Thank you for staying to answer my questions and provide feedback on your experience of the EXTOD Education pilot. I am going to start with some general questions and then there will be some that are more specific.**

**Overall session feedback**

- What did you think of the sessions?
- Did the sessions meet your expectations? (Yes/no – in what way? Please give examples)
- How did you feel about the number of sessions and the spread of these over 6 weeks?
- How did you feel about the length and timing of the sessions?
- What key messages did you take away from the programme?
- What are your thoughts about the group size now that you have experienced the programme?
- Was it helpful having the option to bring someone with you?

**Session specific**

- How did you find session 1?
- What did you think of the resources that supported the content? (Norm and Tod; cycle ride; aerobic/anaerobic exercise; hypo game; traffic light game; High/Low GI; Strategies Pie Chart)
- How did you find the handbook? What changes would you make to it?
- During the first session, a Doctor came to talk to you, how did you find that?
- How important is it to have a Doctor as part of the sessions? What content would you like them to cover?
- How did you find the content in sessions 2 and 3?
- What, if anything, did you feel was missing from the 3 sessions?

**Improvements/developments**

- How would you feel about having a practical element to the programme, for example, taking part in a brisk 30-minute walk?
- If we were to develop an online-resource to accompany the face to face sessions, what should we consider? What should we include?
- *What advice do you have for us to promote the study and the education programme?*

***Your management/impact***

- *Has anything changed in how you manage exercise and your diabetes since attending ExTOD? If so, what are you doing differently?*
- *What role has ExTOD had in making these changes? Would you have made them without coming?*

**Thank you for your time and input. Your comments are invaluable to the development process.**
