# Supplementary material for: Development of a group structured education programme to support safe exercise in people with Type 1 diabetes: the EXTOD education programme
Source: Diabet Med. 2019 Jul 31;37(6):945–52. doi: 10.1111/dme.14064 (PMC7317834; doi:10.1111/dme.14064)
Supplement: Supplementary file 3 — Appendix S2. EXTOD– educator feedback. [file DME-37-945-s003.docx]

**Appendix 2:** Educator feedback form

**We would appreciate your feedback as an educator.**

**When considering your answers, please give as much detail as possible and reflect on your experience of all 3 sessions.**

| **Overall session feedback** |
| --- |
| **How prepared did you feel to deliver the sessions?** |
| **Is there anything else that would have helped you prior to delivering the sessions?** |
| **How confident did you feel to deliver:**   1. **The content?** 2. **Facilitate and manage the group?** |
| **What are your thoughts on the group size?** |
| **How did you find the length and timing of the 3 sessions?** |
| **What would you change about the education programme?** |
| **Who do you think should deliver this programme in the future?** |

Educator feedback form- continued

| **Session-specific feedback** |
| --- |
| **What went well?**   1. **Session 1** 2. **Session 2** 3. **Session 3** |
| **Did you feel you had enough time to deliver your content? (If no, specify which sections you felt needed more or less time?)** |
| **Was anything missing from the sessions?** |
| **What further training would you like to deliver EXTOD education in future?** |
| **What are your thoughts about the handbook/resources/curriculum?**  **How did they support your delivery of the programme?** |
| **Any other comments/feedback** |
